# Twelve-year survival after multiple recurrences and repeated metastasectomies for renal cell Carcinoma

**DOI:** 10.1186/1477-7819-9-155

**Published:** 2011-11-28

**Authors:** Jue Wang, Geoffrey A Talmon, Michael Feloney, Michael C Morris

**Affiliations:** 1 Department of Internal Medicine, Section of Oncology-Hematology, University of Nebraska Medical Center, Omaha, Nebraska 68198-7680, USA; 2 Department of Pathology and Microbiology, University of Nebraska Medical Center, Omaha, Nebraska 68198-3135, USA; 3 Urologic Surgery Section, Department of Surgery, University of Nebraska Medical Center, Omaha, NE 68198-2360, USA; 4 Transplant Surgery Division, Department of General Surgery, University of Nebraska Medical Center, Omaha, NE, USA

**Keywords:** Renal cell carcinoma (RCC), Recurrence, Metastatic disease, Metastasectomy

## Abstract

**Background:**

Metastatic renal cell carcinoma (RCC) presents a therapeutic challenge for clinicians because of the unpredictable clinical course, resistance to chemotherapy or radiotherapy and the limited response to immunotherapy.

**Patients and Methods:**

We report a case of a 62-year-old woman who underwent nephrectomy for T4N0 RCC, clear cell type, Fuhrman grade 3/4 in 1999. The patinet subsequently had multiple tumor recurrences.

**Results:**

The patient underwent eight metastasectomies, including multiple partial left nephrectomies, right adrenalectomy, a complete left nephrectomy, and distal pancreatectomy. She remains well and tumor free 12 years after initial diagnosis.

**Conclusion:**

Repeated resections after initial metastasectomy can be carried out safely and provide long-term survival in selected patients with recurrent metastasis from RCC. The findings from our case indicate that close follow-up for the early detection of recurrence and complete resection of metastases can improve the results after repeated resection.

## Background

Renal cell carcinoma (RCC) accounts for 3% of adult malignancies and 90% of neoplasms arising from the kidney [1]. It is the sixth leading cause of cancer death in the USA [2]. Approximately one-third of patients diagnosed with RCC in the modern era are found to have metastatic disease upon presentation, while at least an additional one-third of all patients undergoing nephrectomy for apparent clinically localized disease will go on to develop metastatic disease [3,4]. The median time before a relapse after nephrectomy is 15 months, and 85% of relapses occur within 3 years [5]. Frequent sites include the lungs (75% of cases), regional lymphatic nodes (65%), bone (40%), liver (40%) and brain (5%) [4]. Unusual sites of metastases can be involved, including the thyroid, pancreas, skeletal muscle and skin or underlying soft tissue. Untreated patients with metastatic RCC have a median survival of 6 to 12 months and a 5-year survival rate of < 20%. Shorter interval between nephrectomy and the development of metastases is associated with a poorer prognosis [4]. Late tumor recurrence occasionally occurs many years after initial treatment.

The role of metastasectomy for the treatment of metastasis from RCC is widely accepted [6]. However, no consensus has been reached concerning the optimal treatment strategies for patients that have already undergone previous metastasectomy, and are later found to have recurrent metastasis. Furthermore, no standard has been proposed that enables adequate answers to questions frequently encountered clinical conditions regarding the benefits actually conferred by repeated resection under the following circumstances: (i) recurrence found in different sites after initial metastasectomy, (ii) the feasibility of a third or even fourth resection of metastasis.

Here we report a patient with multiple recurrences (including ipsilateral adrenal gland, contralateral kidney and pancreas metastasis) after initial nephrectomy, whom was successfully treated with repeated metastasectomies. To our knowledge, this is the first report of such a case. We review current literature on the role of metastasectomy on management of metastatic RCC.

## Case Presentation

**Figure 1:**
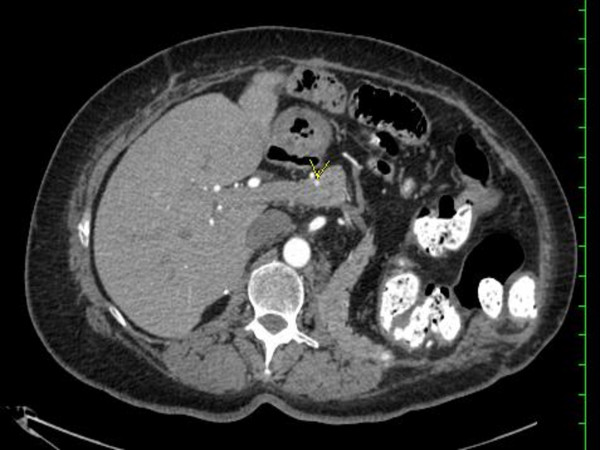
**Radiological evaluation**. A computed tomography of the chest and abdomen demonstrated multiple small enhancing nodules throughout the pancreas.

**Figure 2:**
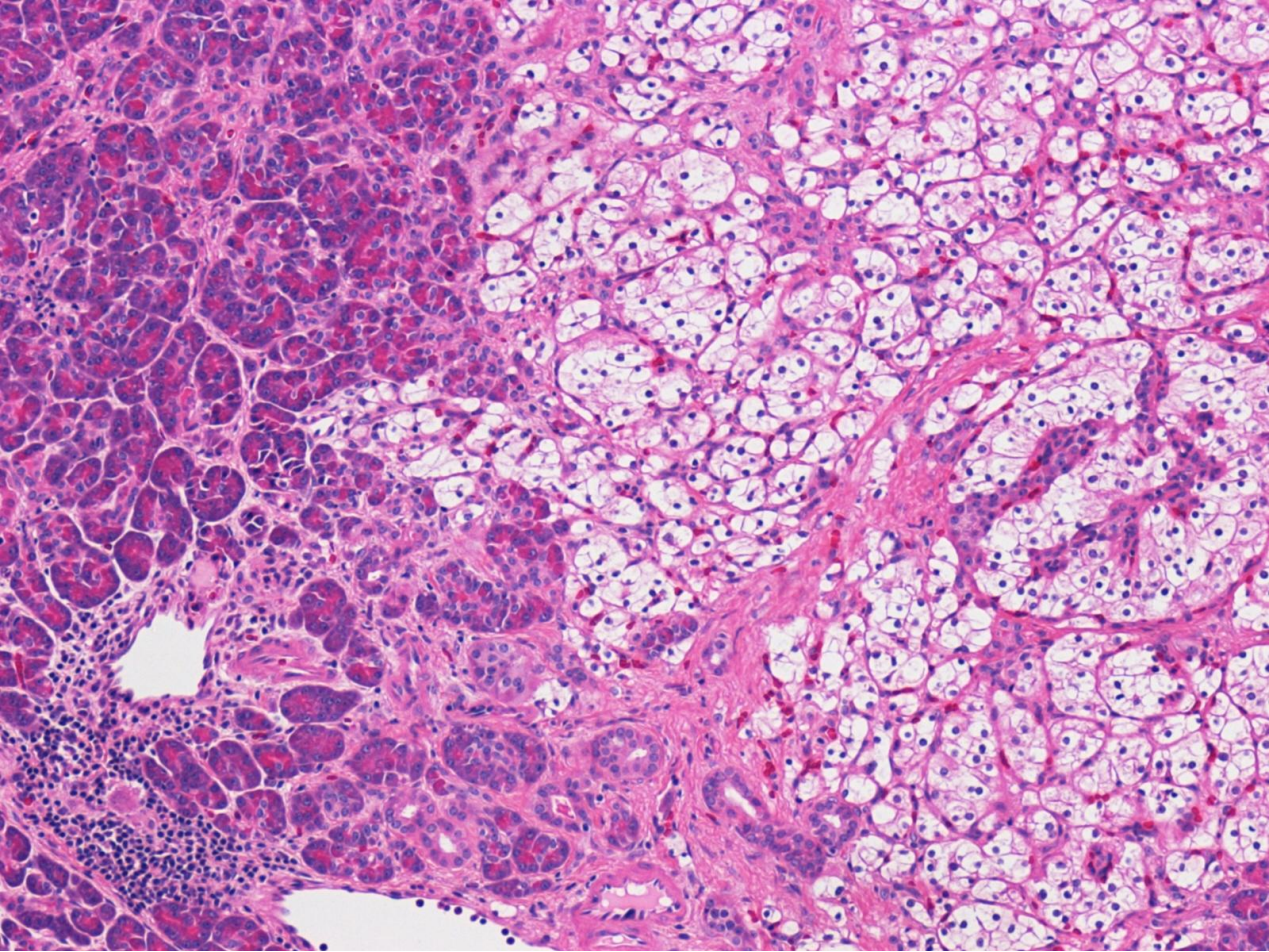
**Histopathological evaluation**. A histological examination of the distal pancreas from pancreatectomy showing metastatic renal cell carcinoma, clear cell type. A high-power view (Hematoxylin and eosin, 100×) showed tumor cells are characterized by epithelial cells with clear cytoplasm and a well-defined cell membrane, interspersed within a highly vascularized stroma.

62-year-old Caucasian female underwent right nephrectomy for T4N0 renal cell carcinoma, clear cell type, Fuhrman grade 3/4 in 1999. In January 2001, a CT scan revealed a right adrenal mass and multiple left side kidney masses. She underwent left partial nephrectomy and right adrenalectomy. At that time, the surgical pathology showed renal cell carcinoma, clear cell type, Fuhrman grade 3/4. The tumors were located in the upper and lower portion of the left kidney. The surgical margin at the upper portion kidney was positive. Right adrenal masses were also excised which revealed adrenal gland with metastatic renal cell carcinoma, clear cell type. Surgical margin of adrenalectomy was negative. The patient subsequently had repeated left partial nephrectomy performed in 2004, and again in 2007 for tumor recurrences. The pathology revealed metastatic renal cell carcinoma. On January 27, 2010, the patient underwent left kidney radical nephrectomy, which revealed multifocal renal cell carcinoma, clear cell type, Fuhrman grade 3/4. The largest tumor nodule was 2.5 cm, with tumor extended focally to renal sinus adipose tissue. Tumor located in 0.1 cm from the nearest soft tissue resection margin. The patient was subsequently started on hemodialysis after surgery. In May 2010, the patient underwent evaluation for possible kidney transplant. During the workup, an ultrasound of the abdomen showed a hypoechoic nodule in the body of the pancreas. A CT scan revealed multiple small enhancing nodules throughout the pancreas, best seen on arteriography, which suspicious for metastatic disease of the pancreas (Figure 1). On September 20 2010, the patient underwent distal pancreatectomy. Histologically, the tumor consisted of cells arranged in trabecular and alveolar structures with clear or eosinophilic granular cytoplasm, compatible with a metastatic RCC, clear cell type, involving pancreatic parenchyma (Figure 2). The surgical margin was negative. Postoperatively, the patient recovered well. She developed recurrent ascites in 2011, which was thought to be related to pancreatic fistula. In July 2011, she underwent exploratory laparotomy, extensive enterolysis, resection of distal pancreatic segment, and repair of enterotomy, chronic pancreatic fistula originating from distal retained pancreatic segment. Surgical pathology revealed chronic pancreatitis with focal granulomatous inflammation, multiple lymph nodes examined without any signs of malignancies. At last follow-up in November 2011, she remains in good physical conditions and tumor free.

## Discussion

Metastatic RCC presents a particular therapeutic challenge for clinicians because of its resistance to chemotherapy and radiotherapy as well as its limited response to immunotherapy [3,4]. Recent advances in understanding the molecular biology of RCC led to the development of several targeted agents that showed impressive anti-tumor efficacy and prolongation of progression-free survival. The integration of these drugs into clinical practice has revolutionized the management of RCC. However, it has also created new controversy concerning the necessity, patient selection, and timing of surgery (nephrectomy, metastasectomy).

Surgical intervention in patients with metastatic RCC can occur in two settings [6,7]: (1) cytoreductive nephrectomy: to resect the primary tumor in the face of unresectable metastatic disease prior to the initiation of systemic therapy, or (2) metastasectomy: to render a patient clinically free of all sites of metastases. Reports to date that have demonstrated a role for resection of RCC metastases have included primarily patients with solitary metastases and/or disease in the lungs only [7-9]. The patients with good performance status undergoing nephrectomy and subsequent complete metastasectomy may experience prolonged survival, which could be attributed to a combination of patient selection factors and the surgical resections [7-11]. However, the utility of metastasectomy in patients with multiple metastases in the era of target therapy has not been well defined. We report this unusual case in a female patient who underwent eight operations for recurrent diseases and remained disease-free twelve years after initial diagnosis, highlighting the tremendous heterogeneity in biologic behavior of metastatic RCC and the role of surgery in long term cancer control.

One of the largest studies on metastasectomy from Memorial Sloan-Kettering Cancer Center [8] reported 278 patients undergoing resection of metastatic RCC over a 13-year period. A multivariate analysis identified factors independently predictive of survival were male gender, disease-free interval > 1 year, single metastatic foci (multiple unilateral lung metastases were considered single), and complete metastasectomy. Investigators at the Mayo Clinic reported a series of 41 patients with metastasectomy over a 20-year period and found a 5-year overall survival rate of 31%. This selected group of patients had a prolonged disease-free interval with a median of 27 months, and excluded patients with skeletal, nodal, and spinal cord metastases [9]. In a recent Mayo study of 887 patients who underwent nephrectomy for RCC between 1976 and 2006 and subsequently developed multiple metastatic lesions [10], the impact of complete metastasectomy on survival was evaluated controlling for the timing, location, and number of metastases and for patient performance status. The results indicated that complete resection of multiple RCC metastases may be associated with long-term survival. A review of experience from M. D. Anderson Cancer Center reported 179 (8.5%) of 2100 patients who were known to have metastatic RCC between 1984 and 1997 underwent resection for an apparently solitary metastatic lesion [11]. The overall 5-year survival rate was 29%. Among the 40 patients with recurrent disease after resection of a lung metastasis, 24 (60%) were able to undergo a subsequent resection compared with only 14 (25%) of the 56 patients with recurrent bone metastasis.

The pancreas is an uncommon site of metastasis for renal cell carcinoma, typically occurring years after treatment of the primary tumor. When the metastatic focus is isolated and the tumor can be resected in its entirety, patients can experience excellent 5-year survival rates. In a retrospective review of patients undergoing pancreatic resection for renal cell carcinomas metastatic to the pancreas or periampullary region between April 1989 and May 1999 at the Johns Hopkins Medical Institutions [12], of 10 patients who underwent pancreatic resection for renal cell carcinoma metastases, six underwent pancreaticoduodenectomy and two underwent distal pancreatectomy, whereas the two remaining patients underwent total pancreatectomy for extensive tumor involvement throughout the entire gland. The mean time from nephrectomy for resection of the primary tumor to reoperation for periampullary recurrence was 9.8 years (median 8.5 years). In a retrospective review of Mayo experience of 23 patients (15 men and 8 women) with RCC metastatic to the pancreas [13], the mean interval between resection of the primary RCC and detection of the pancreatic metastases was 116 months (range, 1-295 months). In 18 patients (78%), the pancreatic metastases were diagnosed more than 5 years after nephrectomy, surgical resection was carried out in 11 patients (7 distal and 3 total pancreatectomies and 1 distal pancreatectomy followed 4 years later by total pancreatectomy), with 8 patients alive at a mean follow-up of 4 years, 6 of whom remained free of recurrence. Overall, 12 patients (52%) were alive at a mean of 42 months after diagnosis of metastatic disease.

The development of vascular endothelial growth factor (VEGF) and the mammalian target of rapamycin (mTOR) inhibitors have markedly improved progress free survival in treatment naive and previously treated patients with metastatic RCC. However, the novel therapies are more likely to be cytostatic than cytotoxic, complete and durable response are rare; chronic therapy are required and side effects are unavoidable [14,15]. Although 40-70% of adverse events (AEs) are grade 1 and 2, 10-20% of patients develop grade 3 or 4 AEs requiring dose reductions, drug holidays or treatment discontinuation. In Phase III trials evaluating VEGF and mTOR inhibitors, the most common grade 3 and 4 AEs observed including hypertension, decreased left ventricular ejection fraction, hand-foot-syndrome and myelosuppression [16]. The long-term impact of these side effects is a concern in RCC patients who are living longer and receiving chronic sequential or combination therapy [16,17]. There is a clear need to develop individualized therapy for patients with metastatic RCC. Our case, in this regard, is quite unique. The patient was treated successfully with repeated metastasectomies for multiple recurrences, without systemic therapy during twelve years follow-up.

## Conclusions

In summary, literature and our experiences suggest surgical resection of multiple recurrences may be worthwhile in selected patients, not only as a mean of local control, but also as a strategy for long term disease control, without the burden of long term drug therapy. Further molecular studies are needed to understand the heterogeneity in biologic behavior of metastatic RCC, which in turn may help develop a more rational way to individualize therapy for patients with RCC.

## Abbreviations

RCC: Renal cell carcinoma; CT: computed tomography; VEGF: Vascular endothelial growth factor; mTOR: The mammalian target of rapamycin; AEs: Adverse events.

## Consent

Written informed consent was obtained from the patient for publication of this case report and any accompanying images. A copy of the written consent is available for review by the Editor-in-Chief of this journal.

## Competing interests

The authors declare that they have no competing interests.

## Authors' contributions

JW and MM carried out the study design and writing. GT carried out the pathology studies, MF participated in the drafting the manuscript. All authors read and approved the final manuscript.
